# Possible Role of Correlation Coefficients and Network Analysis of Multiple Intracellular Proteins in Blood Cells of Patients with Bipolar Disorder in Studying the Mechanism of Lithium Responsiveness: A Proof-Concept Study

**DOI:** 10.3390/jcm13051491

**Published:** 2024-03-05

**Authors:** Keming Gao, Marzieh Ayati, Nicholas M. Kaye, Mehmet Koyuturk, Joseph R. Calabrese, Eric Christian, Hillard M. Lazarus, David Kaplan

**Affiliations:** 1 Department of Psychiatry, University Hospitals Cleveland Medical Center, 10524 Euclid Avenue, 12th Floor, Cleveland, OH 44106, USA; josephcalabresemd@outlook.com; 2 Department of Psychiatry, Case Western Reserve University School of Medicine, Cleveland, OH 44106, USA; 3 Department of Computer Science, University of Texas Rio Grande Valley, Edinburg, TX 78539, USA; marzieh.ayati@utrgv.edu; 4 CellPrint Biotechnology LLC, Cleveland, OH 44106, USA; nick.m.kaye@gmail.com (N.M.K.); echristian@cellprintbiotechnology.com (E.C.); hml@case.edu (H.M.L.); dkaplan@cellprintbiotechnology.com (D.K.); 5 Department of Computer and Data Sciences, Center for Proteomics and Bioinformatics, Case Wester Reserve University, Cleveland, OH 44106, USA; mxk331@case.edu; 6 Department of Medicine, University Hospitals Cleveland Medical Center, Cleveland, OH 44106, USA; 7 Department of Medicine, Case Western Reserve University School of Medicine, Cleveland, OH 44106, USA

**Keywords:** lithium, bipolar disorder, monocytes, lymphocytes, biomarkers, correlation coefficient, protein-to-protein interaction network, pathway analysis

## Abstract

**Background:** The mechanism of lithium treatment responsiveness in bipolar disorder (BD) remains unclear. The aim of this study was to explore the utility of correlation coefficients and protein-to-protein interaction (PPI) network analyses of intracellular proteins in monocytes and CD4^+^ lymphocytes of patients with BD in studying the potential mechanism of lithium treatment responsiveness. **Methods:** Patients with bipolar I or II disorder who were diagnosed with the MINI for DSM-5 and at any phase of the illness with at least mild symptom severity and received lithium (serum level ≥ 0.6 mEq/L) for 16 weeks were divided into two groups, responders (≥50% improvement in Montgomery-Asberg Depression Rating Scale and/or Young Mania Rating Scale scores from baseline) and non-responders. Twenty-eight intracellular proteins/analytes in CD4^+^ lymphocytes and monocytes were analyzed with a tyramine-based signal-amplified flow cytometry procedure. Correlation coefficients between analytes at baseline were estimated in both responders and non-responders and before and after lithium treatment in responders. PPI network, subnetwork, and pathway analyses were generated based on fold change/difference in studied proteins/analytes between responders and non-responders. **Results:** Of the 28 analytes from 12 lithium-responders and 11 lithium-non-responders, there were more significant correlations between analytes in responders than in non-responders at baseline. Of the nine lithium responders with pre- and post-lithium blood samples available, the correlations between most analytes were weakened after lithium treatment with cell-type specific patterns in CD4^+^ lymphocytes and monocytes. PPI network/subnetwork and pathway analyses showed that lithium response was involved in four pathways, including prolactin, leptin, neurotrophin, and brain-derived neurotrophic factor pathways. Glycogen synthase kinase 3 beta and nuclear factor NF-kappa-B p65 subunit genes were found in all four pathways. **Conclusions**: Using correlation coefficients, PPI network/subnetwork, and pathway analysis with multiple intracellular proteins appears to be a workable concept for studying the mechanism of lithium responsiveness in BD. Larger sample size studies are necessary to determine its utility.

## 1. Introduction

Lithium can be used as a first-line treatment for bipolar disorder (BD) [1]. In the acute treatment of patients with BD, up to two-thirds of them may have at least 50% improvement from baseline [2,3,4,5]. However, the mechanism of lithium treatment response remains unclear [6,7]. Studies from human induced pluripotent stem cells (iPSC) suggest that bipolar lithium responders and non-responders have identifiable but different intracellular biomarkers and lithium treatment is linked to differential gene expression and electrophysiological activities in responders and non-responders [8,9]. A study from lymphoblastoid cell lines also found that lithium differentially upregulated and downregulated specific genes and gene networks in responders and non-responders [10]. Moreover, the studies from PGBD (Pharmacogenomic Study of Bipolar Disorder) found that lithium treatment response was associated with the architecture of circadian rhythms that were regulated with lithium in responders [11,12]. A network-based multi-omics analysis from the PGBD found that the molecules related to focal adhesion on axon guidance and neuronal circuits were associated with the mechanisms of response to lithium [13].

Although the aforementioned studies suggest that there are potential identifiable biomarkers for lithium treatment responsiveness, the effort in the field has not yielded any biomarker(s) for the use of lithium in routine clinical practice. One of the main limitations of previous studies is that none has used multiple intracellular proteins as potential biomarkers to study the treatment response of lithium in BD. In an effort to study potential protein biomarker(s), especially intracellular proteins, in lithium responsiveness, our group has used a highly sensitive, tyramine-based, signal-amplified flow cytometry developed by the CellPrint Biotechnology (the CellPrint™) [14,15] to study the intracellular proteins in mononuclear blood cells of patients with BD who received lithium treatment.

In our first analysis [16], we included 28 analytes in CD4^+^ lymphocytes and monocytes of the blood samples from lithium responders and non-responders at baseline and at the end of the study. The 28 analytes were BAX (BAX, BCL2-Associated X Protein), BCL-2 (B-cell lymphoma 2), BCL-2 A1 (Bcl-2-related protein A), BDNF (brain-derived neurotrophic factor), CALM1 (calcium-modulated protein 1), Fyn (a tyrosine kinase belongs to the Src family of tyrosine kinases including src, fyn, and yes), GSK3β (glycogen synthase kinase 3 beta), HMGB1 (high mobility group box 1 protein), iNOS (inducible isoform nitric oxide synthases), IRS2 (insulin receptor substrate 2), MARCKS (myristoylated alanine-rich C-kinase substrate), mTor (mammalian target of rapamycin), NLRP3 (NACHT, LRR and PYD domains-containing protein 3), NR3C1 (nuclear receptor subfamily 3, group C, member 1), phospho-CREB (phosphorylated cAMP response element-binding protein), phospho-Fyn/phospho-Yes (phosphorylated Fyn/phosphorylated Yes), phospho-GSK3α/β (phosphorylated glycogen synthase kinase 3 α/β), phospho-GSK3β (phospho-glycogen synthase kinase 3 beta), phospho-NFkB-P65 (phosphorylated nuclear factor NF-kappa-B p65 subunit), PDEB4 (cAMP-specific 3′,5′-cyclic phosphodiesterase 4B), PGM1 (phosphoglucomutase 1), PKA C-α (protein kinase A catalytic subunit alpha), PKC-θ (protein kinase C theta), PPAR-γ (peroxisome proliferator-activated receptor gamma), Timeless (a protein is necessary of proper functioning of circadian rhythm), TNFAIP3 (tumor necrosis factor, alpha-induced protein 3), TPH1 (tryptophan hydroxylase 1), and XBP1 (X-box binding protein 1). These proteins are involved in apoptosis (BAK, Bcl-2,Bcl-2 A1)**,** calcium transport (CALM1), cell signaling (GSK3β, phospho-GSK3α/β, phospho-GSK3β, iNOS, PDEB4)], circadian rhythm (timeless), metabolic enzymes (PGM1, TPH1), inflammation (HMGB1, NLPR3, TNFAIP3, MARCKS), kinase (phospho-Fyn/phospho-Yes, PKA C-α, PKC-θ, mTor), neurotrophic factors (BDNF), receptors (IRS2, PPAR-γ, NR3C1), transcriptional factors (phospho-NFkB-P65, phospho-CREB, XBP1). The selection of these analytes was based on previous studies [17,18,19,20,21,22,23,24,25,26,27,28] and possible mechanisms of lithium treatment response [7].

Comparing these analytes at baseline, we found that lithium non-responders had higher but insignificant levels of the majority of analytes in both cell types than responders. Comparing these analytes before and after lithium treatment, the levels of most analytes in lithium responders increased after lithium, with some reaching significantly different levels compared to before lithium. In contrast, the levels of most analytes in lithium non-responders were decreased, with some reaching significantly different levels after lithium treatment compared to before lithium [16].

In our second analysis [29], we included 17 analytes of the initial 28 analytes in CD4^+^ lymphocytes and monocytes of the blood samples from the lithium responder and non-responders at baseline only. The selection of the 17 analytes was mainly based on the results of the initial analysis [16]. Of the 17 analytes in lithium responders and non-responders, we also found that lithium non-responders had higher levels of analytes at baseline than responders in both cell types. In addition, some analytes were able to predict treatment outcomes. For instance, using baseline GSK3β and phosphorylated GSK3αβ levels in monocytes, we were able to correctly classify eleven of eleven (100%) responders and five of eight (63%) non-responders. Using baseline levels of GSK3β, phosphorylated NFkB-P65, TPH1, and PGM1, we were able to correctly classify ten of eleven (91%) responders and six of seven (96%) non-responders, both with a probability of correct classification of ≥85%.

Our protein-protein interaction (PPI) network and pathway analyses from the second analysis included 14 proteins related to the 17 analytes. These 14 proteins were BCL2, BDNF, CALM1, GSK3β, HMGB1, IRS2, mTOR, NLPR3, iNOS1, PGM1, PPAR-γ, PKA C-α, NFkB-P65, and TPH1 and they were in a network of a total 71 proteins. The expression levels of those 14 proteins in both CD4^+^ lymphocytes and monocytes significantly overlapped. Our pathway analysis found that prolactin, leptin, BDNF, neurotrophin, and EGF (epidermal growth factor)/EGFR (epidermal growth factor receptor) signaling pathways were involved in the network of proteins in the study.

Taken together, our previous analyses found that lithium non-responders had higher levels of most studied intracellular proteins in both cell types than lithium responders. Lithium treatment increases protein levels in lithium responders and decreases the same protein levels in non-responders. PPI network analysis suggests that lithium responsiveness may be involved with multiple proteins, but some proteins may play a more important role than others. Clearly, it remains unclear how these proteins interact with each other at baseline and after lithium treatment in lithium responders and non-responders. Understanding the patterns of PPI before and after lithium in responders and non-responders may help us understand the underlying mechanism of lithium responsiveness.

In the current proof-concept study, we used the same initial 28 intracellular analytes of both responders and non-responders at baseline and of responders before and after lithium treatment to elucidate interactions between analytes by using a correlation coefficient. However, it should be kept in mind that correlation is not causation, so we would be unable to use correlation coefficients between analytes to determine the expression levels of any proteins/analytes in our previous analysis [16,29]. We also conducted PPI network and pathway analyses as we did previously [29] and PPI subnetwork analyses on specific pathways. In doing so, we may be able to provide information on the usefulness of conducting correlation, PPI network/subnetwork, and pathway analyses to study potential targets that may be involved in lithium treatment responsiveness for future in vivo and in vitro studies. The abstract of these analyses was presented at the 2023 Annual Meeting of the International Society for Bipolar Disorders [30].

## 2. Methods

### 2.1. Study Design

The study design, participants, study procedures, and blood collection were described previously [16,29]. Briefly, this study was an open-label, 16-week study of lithium monotherapy in patients with BD (bipolar I or II disorder) who were in depression or mania/hypomania with or without mixed features and with at least mild symptoms (clinicaltrial.gov, NCT02909504, https://classic.clinicaltrials.gov, accessed on the 25 February 2024). The study was conducted in accordance with the Declaration of Helsinki and approved by the Institutional Review Board of the University Hospitals Cleveland Medical Center (UHCMC IRB number: 07-16-05) on 7 December 2016. Written informed consent was obtained before any study procedure proceeded.

The psychiatric diagnoses were conducted with the Mini International Psychiatric Interview for DSM-5 and a structured diagnostic interview for research. Symptom severity was measured with standardized rating scales for depression (Montgomery-Asberg Depression Rating Scale, MADRS), anxiety (Hamilton Anxiety Rating Scale), or mania (Young Mania Rating Scale (YMRS). Disability was measured with the Sheehan Disability Rating Scale (SDS), and the quality of life was measured with the Quality of Life, Enjoyment, and Satisfaction Questionnaire (Q-LES-Q). Rating scales were completed at baseline and then at week 1, week 2, week 4, week 6, week 8, week 12, and week 16. Eligible patients who received lithium treatment for up to 16 weeks. However, unpermitted ongoing medications were tapered off by week 4 per protocol. Blood samples of all patients were collected at the baseline and at the end of 16 weeks or at the time of early termination. Intracellular proteins in monocytes and CD4^+^ lymphocytes of the responders and non-responders were analyzed with the CellPrint™ after the study was completed.

### 2.2. Antibodies and Cytometric Analyses

The procedure of cytometric analyses was described previously [16,29]. Briefly, the separation of CD4^+^ lymphocytes and monocytes from the rest of the blood components was employed using manufacturer-standard protocols. Amplified signals for 28 intracellular analytes were generated with commercially available antibodies to the target proteins or phospho-proteins. The names and functions of the 28 analytes were described previously [16] as well as in the Introduction. Once these primary antibodies were bound with the targeted analytes, the peroxidase enzyme was bound to the primary antibodies through secondary antibodies. We used a tyramide-fluorophore conjugate substrate for peroxidase to amplify the signals. Fluorescence levels of the analytes were acquired with a BD Accuri C6 flow cytometer (BD Biosciences, Franklin Lakes, NJ, USA). The median fluorescence intensity (MFI) of each analyte was recorded as the strength of signals for each analyte. Normalization of MFI for each analyte was performed to use the fluorescence minus one (FMO) control to generate a median fluorescence ratio (MFR) that is a quantitative measure of relative protein expression level. MFR = 1 indicated that no signal was detected for an analyte.

After completing the flow cytometric analyses, the raw data of analytes were sent to the data management statistical analysis unit of the Mood Disorders Program. The clinical research team provided the code for each subject (responders or non-responders) to the statistics team. The team linked the code to raw data that were used to compare differences between responders and non-responders.

### 2.3. Raw Data Normalization

The MFR (raw data) of each analyte was further normalized with fold change/difference (FC) of the analyte between responders and non-responders. The FC of each analyte was calculated with log_2_$\left(\frac{the\;average\;of\;MFR\;of\;Responders}{the\;average\;of\;MFR\;of\;\mathrm{Non-Responders}}\right)$. The value of FC was an indicator of the magnitude of the difference of an analyte between lithium responders and non-responders. A positive FC is indicative of a higher expression level in responders relative to non-responders, and a negative FC is indicative of a lower expression level in responders relative to non-responders. In the current study, we used the FC of each analyte between responders and non-responders to generate a PPI network and subnetworks.

### 2.4. Correlation Analysis

The correlation coefficient between two analytes was estimated with simple linear regression and presented with R squared. The MFR of each analyte was used for correlation analysis. For each analyte at baseline, the correlation coefficient with the other 27 analytes was estimated in lithium responders and non-responders, respectively. In lithium responders, the correlation coefficient between GSK3β, PDEB4, and NLRP3 and the other 27 analytes before and after lithium treatment was estimated. However, the correlation coefficient of analytes before and after lithium in non-responders was not estimated because only four non-responders had pre- and post-lithium blood samples. The rationale for studying the correlation between GSK3β and other analytes in lithium responders in the current study was based on the fact that GSK3β is the most studied protein in lithium treatment of BD [7]. For PDEB4, the level of PDEB4 was significantly increased with lithium treatment in both lymphocytes and monocytes in lithium responders [16]. The level of NLRP3 did not increase significantly after lithium treatment in lithium responders in either cell type [16], but NLRP3, as well as HMGB1, TNFAIP3, and MARCKS, plays an important role in inflammation. A number of studies have showed the level of inflammation may affect lithium treatment response in BD [18,28,31,32,33,34,35]. For this reason, we selected the correlation between NLRP3 and other analytes to explore the changes in interactions between proteins after lithium treatment in addition to GSK3β and PDEB4.

### 2.5. Statistical Analysis

The statistical method was used depending on the nature of the variables [16,29]. Categorical variables were analyzed with Chi-square or Fisher Exact tests, and continuous variables were analyzed with T-test. The response was defined as a reduction of ≥50% MADRS and/or YMRS total scores from baseline to the end of the study. The flow cytometric data of each of the 28 analytes in both cell types between two groups at baseline were analyzed with an unpaired *t*-test. However, the differences in the 28 analytes within the response group before and after lithium treatment were analyzed with paired *t*-tests [16]. Because this study was an explorative and hypothesis-generating study, we did not adjust for multiple comparisons in any analysis.

### 2.6. Protein-to-Protein Interaction and Pathway Analysis

The FCs of all proteins in monocytes and CD4^+^ lymphocytes of responders and non-responders at baseline were used for PPI network analysis. We used the same procedures as previously described to generate a PPI network [29]. The rationale for using PPI network analysis to study the phenotypes of lithium responsiveness in the current study was mainly based on the fact that the proteins involved in similar phenotypes are clustered together in cellular networks, and those cellular networks could be revealed with network-based analysis of diverse phenotypes [36]. For PPI network analysis, we used network propagation algorithms [37] to identify the highly connected modules (functional modules). Those modules are commonly in the center of the studied proteins that can be used as the seeds for propagation [29]. For every pair of proteins in the study, we used the shortest path to compute the distance between the proteins. We used random walks with restart at a seed to generate networks. The BioGRID database was used to generate the PPI network and subnetworks. There are 8839 proteins and 67,056 interactions in the BioGRID database [38]. After the pathways were identified with pathway enrichment analysis, the PPI subnetworks were generated.

Pathway enrichment analysis on the induced PPI networks was conducted after the modules were identified. For this purpose, a hypergeometric model was used to assess the significance of the pathways in the Wiki Pathways dataset. We used the Enrichr R package (v2.1) [39] to conduct pathway enrichment analysis.

## 3. Results

### 3.1. Demographics, Historical Correlates, and Change in Symptom Severity

The demographics, historical and clinical correlates, and treatment response to lithium of the 25 patients were published [29]. Of the 25 patients, twenty-four met our intent-to-treat (ITT) sample. These 24 patients returned for at least one post-baseline visit after receiving lithium treatment. Of the 24 patients, 13 were lithium responders, and 11 were lithium non-responders. There were no significant differences in expression levels of 28 analytes at baseline between responders and non-responders [16].

### 3.2. Correlation Coefficient of 28 Analytes at Baselineresponders vs. Non-Responders

**Figure 1:**
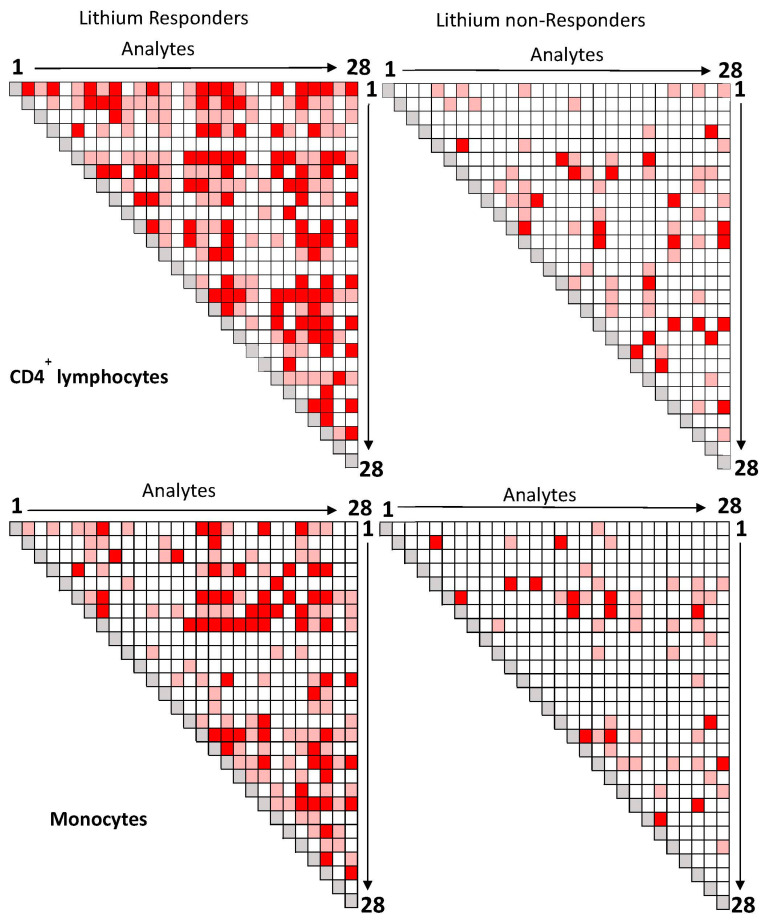
Correlations between 28 analytes in CD4^+^ lymphocytes and monocytes of lithium responders and non-responders at baseline. Legends: Each square represents the magnitude of the correlation coefficient between two analytes (Red: *p* < 0.01; Pink: *p* ≤ 0.05; White: *p* > 0.05; Gray: r = 1). The order of analytes from 1 to 28 is: NRLP3, PDEB4, BAK, phospho-Fyn/phospho-Yes, HMGB1, BCL-2A1, BDNF, GSK3β, XBP1, MARCKS, phospho-GSK3β, phospho-GSK3αβ, Fyn, Timeless, PGM1, phospho-NFkB-P65, Calmodulin, IRS2, phospho-CREB, PPAR-*γ*, TNFAIP3, TPH1, PKC-θ, BCL-2, iNOS, NR3C1, mTor, PKA C-α. Abbreviations: BAX: BAX, BCL2-Associated X Protein; BCL-2: B-cell lymphoma 2; BCL-2 A1: Bcl-2-related protein A1; BDNF: brain-derived neurotrophic factor; Calmodulin: calcium-modulated protein; Fyn: a tyrosine kinase belongs to the Src family of tyrosine kinases including src, fyn, and yes; GSK3β: glycogen synthase kinase 3 beta; HMGB1: High mobility group box 1 protein; iNOS: inducible isoform nitric oxide synthases; IRS2: Insulin receptor substrate 2; MARCKS: myristoylated alanine-rich C-kinase substrate; mTor: mammalian target of rapamycin; NLRP3: NACHT, LRR and PYD domains-containing protein 3; NR3C1: nuclear receptor subfamily 3, group C, member 1; phospho-CREB: phosphorylated cAMP response element-binding protein (Ser133); phospho-Fyn/phospho-Yes: phosphorylated Fyn (Y530)/phosphorylated Yes (Y537); phospho-GSK3α/β: phosphorylated glycogen synthase kinase 3 alpha (Tyr279) beta (Tyr216); phospho- GSK3β: phospho-glycogen synthase kinase 3 beta (Tyr216); phospho-NFkB-P65: phosphorylated nuclear factor NF-kappa-B p65 (Ser536) subunit; PDEB4: cAMP-specific 3′,5′-cyclic phosphodiesterase 4B; PGM1: phosphoglucomutase 1; PKA C-α: protein kinase A catalytic subunit alpha; PKC-θ: protein kinase C theta; PPAR-*γ*: peroxisome proliferator-activated receptor gamma; Timeless: a protein is necessary of proper functioning of circadian rhythm; TNFAIP3: tumor necrosis factor, alpha-induced protein 3; TPH1: tryptophan hydroxylase 1; XBP1: X-box binding protein 1.

The blood samples of twelve of thirteen responders and all eleven non-responders were available for baseline correlation analysis of 28 analytes. As shown in Figure 1, the number of significant correlations between analytes in CD4^+^ lymphocytes (upper panel) and monocytes (lower panel) in lithium responders (left panels) was more than that compared to lithium non-responders (right panels).

In responders, a number of significant correlations between the same two analytes were observed in both CD4^+^ lymphocytes (upper panel) and monocytes (lower panel), but most correlations between the same two analytes were cell-type specific. In lithium non-responders (right panels), only a few significant correlations between the same two analytes were observed in both cell types, and most correlations between two analytes were also cell-type specific.

### 3.3. Correlation Coefficients between GSK3β and Other Analytes in Responders before and after Lithium Treatment

**Figure 2:**
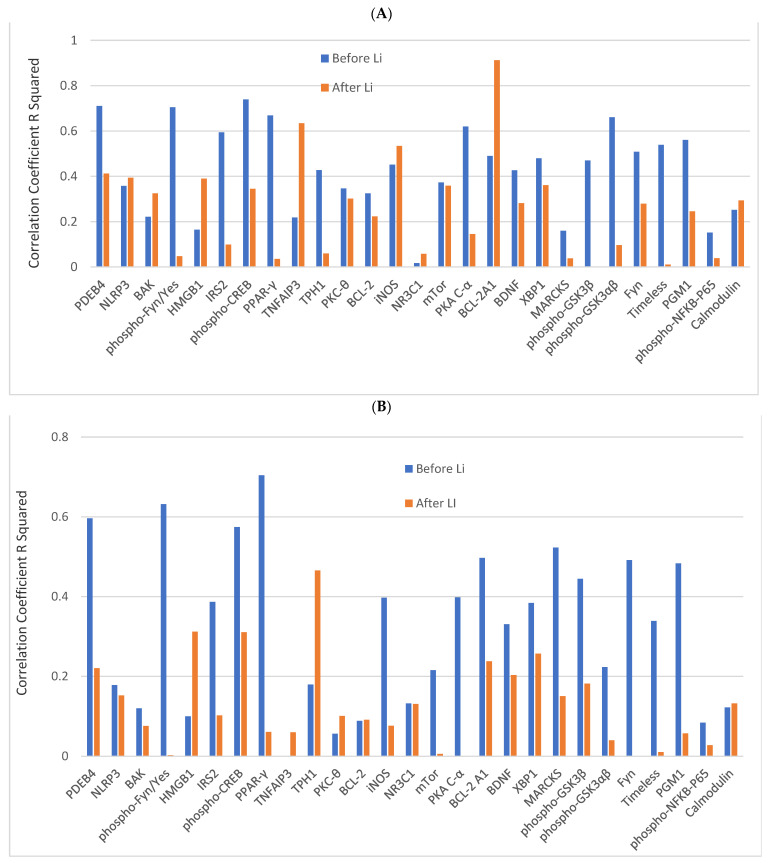
The correlation coefficients R Squared between GSK3β and other analytes in CD4^+^ lymphocytes and monocytes of responders before and after lithium treatment. Legends: (**A**) is the correlation coefficient R Squared between GSK3β and other analytes in CD4^+^ lymphocytes, and (**B**) is the correlation coefficient R Squared between GSK3β and other analytes in lymphocytes. The blue bar is before lithium, and the orange bar is after lithium. Abbreviations: BAX: BAX, BCL2-Associated X Protein; BCL-2: B-cell lymphoma 2; BCL-2 A1: Bcl-2-related protein A1; BDNF: brain-derived neurotrophic factor; Calmodulin: calcium-modulated protein; Fyn: a tyrosine kinase belongs to the Src family of tyrosine kinases including src, fyn, and yes; GSK3β: glycogen synthase kinase 3 beta; HMGB1: High mobility group box 1 protein; iNOS: inducible isoform nitric oxide synthases; IRS2: Insulin receptor substrate 2; MARCKS: myristoylated alanine-rich C-kinase substrate; mTor: mammalian target of rapamycin; NLRP3: NACHT, LRR and PYD domains-containing protein 3; NR3C1: nuclear receptor subfamily 3, group C, member 1; phospho-CREB: phosphorylated cAMP response element-binding protein (Ser133); phospho-Fyn/phospho-Yes: phosphorylated Fyn (Y530)/phosphorylated Yes (Y537); phospho-GSK3α/β: phosphorylated glycogen synthase kinase 3 alpha (Tyr279) beta (Tyr216); phospho- GSK3β: phospho-glycogen synthase kinase 3 beta (Tyr216); phospho-NFkB-P65: phosphorylated nuclear factor NF-kappa-B p65 (Ser536) subunit; PDEB4: cAMP-specific 3′,5′-cyclic phosphodiesterase 4B; PGM1: phosphoglucomutase 1; PKA C-α: protein kinase A catalytic subunit alpha; PKC-θ: protein kinase C theta; PPAR-*γ*: peroxisome proliferator-activated receptor gamma; Timeless: a protein is necessary of proper functioning of circadian rhythm; TNFAIP3: tumor necrosis factor, alpha-induced protein 3; TPH1: tryptophan hydroxylase 1; XBP1: X-box binding protein 1.

The correlation between GSK3β and other analytes in lithium responders before and after lithium treatment *(n* = 9) is presented in Figure 2. In CD4^+^ lymphocytes, before lithium, the correlation coefficients between GSK3β and other analytes varied widely (blue bars in Figure 2A). The levels of 13 analytes had significant correlations with GSK3β with a *p*-value of ≤0.05. Of the 13 analytes, 6 analytes (PDEB4, phospho-CREB, phospho-GSK3α/β, PKA C-α, PPAR-γ, phospho-Fyn/phospho-Yes) had a *p*-value of ≤0.01. After lithium, the correlation coefficients between GSK3β and other analytes also varied widely (brown bars in Figure 2A). The levels of three analytes (TNFAIP3, iNOS, and BCL2 A1) were significantly correlated to the level of GSK3β with a *p*-value of ≤0.05, and two analytes (TNFAIP3 and BCL2 A1) with a *p*-value of ≤0.01.

In monocytes, before lithium, the correlation coefficients between GSK3β and other analytes also varied widely (blue bars in Figure 2B). The levels of 9 analytes had significant correlations with GSK3β with a *p*-value of ≤0.05, and 4 of them (phospho-CREB, PKA-Cα, PPAR-γ, phospho-Fyn/phospho-Yes) had a *p*-value of ≤0.01. After lithium, the correlation coefficients between GSK3β and other analytes also varied widely (brown bars in Figure 2B). Only one analyte (TH1) was still significantly correlated to the level of GSK3β with a *p*-value of ≤0.05.

### 3.4. Correlation between PDEB4 and NLRP3 and Other Analytes before and after Lithium Treatment in Responders

The correlation coefficients between PDEB4 or NLRP3 and other analytes also varied widely before and after lithium in both cell types. In lymphocytes, before lithium, nineteen analytes had significant correlations with PDEB4 at a *p*-value of ≤0.05 and 10 analytes at a *p*-value of ≤0.01. After lithium, 12 analytes had significant correlations with PDEB4 at the *p*-value of ≤0.05 and 5 analytes at the *p*-value of ≤0.01 (see supplemental text and Figure S1A). In monocytes, before lithium, 10 analytes had significant correlations with PDEB4 at a *p*-value of ≤0.05 and 8 analytes at a *p*-value of ≤0.01. After lithium, four analytes had a significant correlation with PDEB4 at the *p*-value of ≤0.05, but none was at the *p*-value of ≤0.01 (see supplemental text and Figure S1B).

The correlation between NLRP3 and other analytes showed a similar pattern before and after lithium in both cell types. In CD4^+^ lymphocytes, before lithium (fifteen analytes had significant correlations with NLRP3 at a *p*-value of ≤0.05 and 9 analytes at a *p*-value of ≤0.01. After lithium, two analytes had significant correlations with NLRP3 at a *p*-value of ≤0.05, but none at the level of the *p*-value of ≤0.01 (see supplemental text and Figure S2A). In monocytes, before lithium, seven analytes had significant correlations with NLRP3 at a *p*-value of ≤0.05 and one analyte at a *p*-value of ≤0.01. After lithium, two analytes had significant correlations with PDEB4 at the *p*-value of ≤0.05, but none was at the *p*-value of ≤0.01 (see supplemental text and Figure S2B).

### 3.5. Protein-Protein Interaction Network Analysis

**Figure 3:**
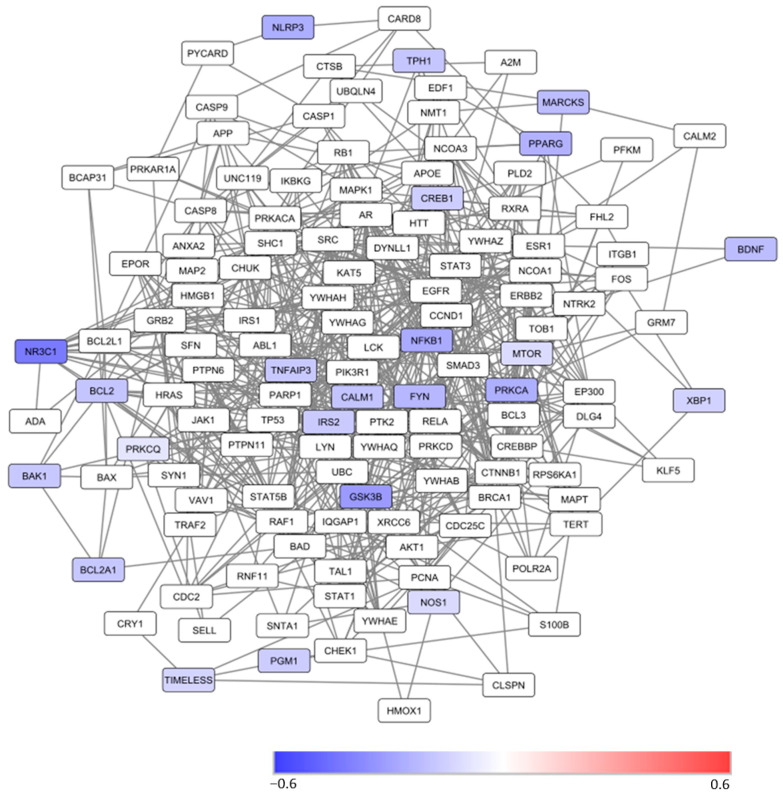
A protein-protein interaction network of 23 studied proteins related to the 28 analytes in CD4^+^ lymphocytes that are mapped to the BioGRID database. Note: The color of the nodes represents the magnitude of differences in protein expression levels between lithium responders and non-responders at the baseline as measured with the $\log_{2}\left(\frac{the\;average\;of\;MFR\;of\;Responders}{the\;average\;of\;MFR\;of\;\mathrm{Non-Responders}}\right)$. A positive value is indicative of a higher level of protein expression in lithium responders relative to non-responders. A negative value is indicative of a lower level of protein expression in lithium responders relative to non-responders. The white nodes are proteins that were not measured in the current study, but they are on the shortest paths between pairs of proteins that are included in the study. PDEB4 was not in the network; phospho-GSK3β, GSK3β, and phospho-GSK3αβ = GSK; fyn and phospho-fyn/yes = FYN. Abbreviations: BAX1: BAX, BCL2-Associated X Protein; BCL2: B-cell lymphoma 2; BCL2A1: Bcl-2-related protein A1; BDNF: Brain-derived neurotrophic factor; CALM1: calcium-modulated protein; CREB1: phosphorylated cAMP response element-binding protein; FYN: a tyrosine kinase belongs to the Src family of tyrosine kinases including src, fyn, and yes; GSK3B: Glycogen synthase kinase 3 beta; HMGB1: High mobility group box 1 protein; IRS2: insulin receptor substrate; MARCKS: myristoylated alanine-rich C-kinase substrate; MTOR: Mammalian target of rapamycin; NLPR3: NACHT, LRR and PYD domains-containing protein 3; NOS1: Inducible isoform nitric oxide synthase; NR3C1: nuclear receptor subfamily 3, group C, member 1; PGM1: Phosphoglucomutase 1; PPARG: Peroxisome proliferator-activated receptor gamma; PRKCA: Protein kinase A catalytic subunit alpha; PRKCQ: protein kinase C theta; NFKB1: nuclear factor NFkB p65 subunit; TIMELESS: a protein is necessary of proper functioning of circadian rhythm; TNFAIP3: tumor necrosis factor, alpha-induced protein 3; TPH1: Tryptophan hydroxylase 1.

Of the 28 analytes, there were 23 proteins. The PPI network of these 23 proteins in CD4^+^ lymphocytes and monocytes was generated by using the same methodology as previously described [29]. These 23 proteins are in a network of more than 130 proteins in CD4^+^ lymphocytes (Figure 3) and monocytes (see supplemental text and Figure S3). With the exception of protein kinase C theta (RKCQ) in monocytes (Figure S3), all other studied proteins in the network had a lower level of expression in lithium responders than in nonresponders.

### 3.6. Pathway Enrichment Analysis and Protein-Protein Interaction Subnetwork Analysis

Pathway enrichment analysis in the PPI network of the 23 proteins in the study found that the proteins in the network are involved in many pathways. These pathways included BDNF, prolactin, neurotrophin, and leptin signaling pathways (Table 1). Both *GSK3β* and *NFkB* genes coding these two proteins are involved in all four pathways.

**Figure 4:**
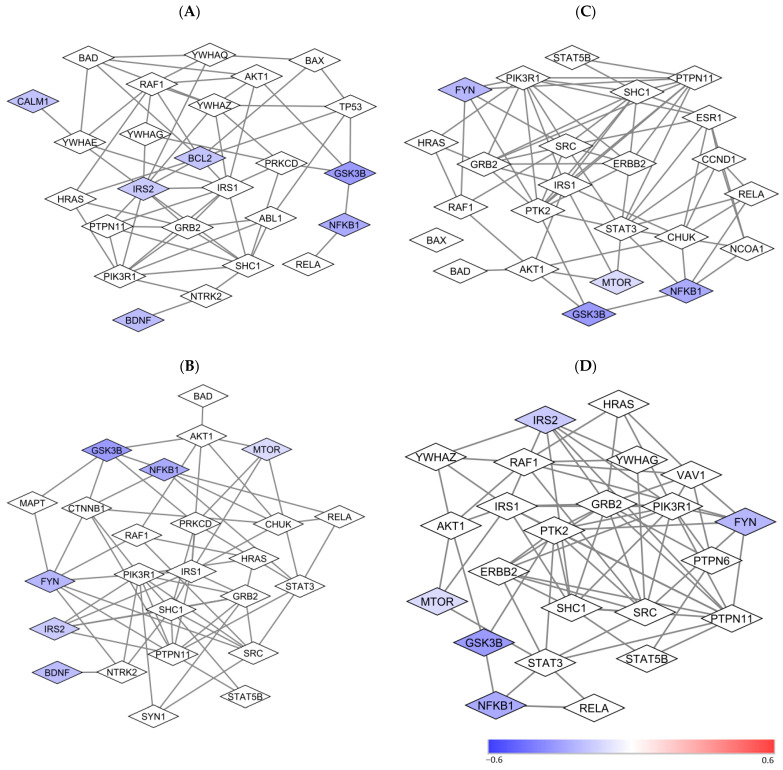
Protein-to-protein subnetworks involved in prolactin, leptin, BDNF, and neurotrophin signaling pathways in CD4^+^ lymphocytes. Note: The color of the nodes represents the magnitude of differences in protein expression levels between lithium non-responders and responders at the baseline as measured with the $\log_{2}\left(\frac{the\;average\;of\;MFR\;of\;Responders}{the\;average\;of\;MFR\;of\;\mathrm{Non-Responders}}\right)$. A positive value is indicative of a higher level of protein expression in lithium responders relative to non-responders. A negative value is indicative of a lower level of protein expression in lithium responders relative to non-responders. The white nodes are proteins that were not measured in the current study, but they are on the shortest paths between pairs of proteins that are included in the study. Abbreviations: BCL2: B-cell lymphoma 2; BDNF: Brain-derived neurotrophic factor; CALM1: calcium-modulated protein; FYN: a tyrosine kinase belongs to the Src family of tyrosine kinases including src, fyn, and yes; GSK3B: Glycogen synthase kinase 3 beta; IRS2: insulin receptor substrate; MTOR: Mammalian target of rapamycin; NFKB1: nuclear factor NFkB p65 subunit. (**A**) Neurotrophin signaling pathway; (**B**) BDNF Signaling Pathway; (**C**) Leptin Signaling Pathway; (**D**) Prolactin Signaling Pathway.

According to the pathway analysis results, PPI subnetworks involved in prolactin, leptin, BDNF, and neurotrophin signaling pathways were generated. Most proteins in lymphocytes (Figure 4) in the subnetwork were not included in the current study. Direct and indirect functional “connections” of the studied proteins with other unstudied proteins were observed.

## 4. Discussion

The aim of this proof-concept study was to explore the utility of using correlation coefficients, PPI network/subnetwork, and pathway analyses of multiple intracellular proteins in CD4^+^ lymphocytes and monocytes to study the mechanism of lithium responsiveness in BD. We found that at baseline, lithium nonresponders had weaker correlations between studied proteins in both CD4^+^ lymphocytes and monocytes than lithium responders (Figure 1). Among the lithium responders, lithium treatments weakened the correlations between 3 selected analytes and most of the other analytes in both cell types, with cell-type specific changes (Figure 2). The PPI network/subnetwork analysis found that the majority of proteins related to lithium treatment response were not included in the current study (Figure 3), and multiple signal pathways might be involved in lithium treatment responsiveness.

Previously, we found that the baseline levels of the most studied analytes in lithium nonresponders were higher than lithium responders [16,29], and baseline levels of some analytes were able to correctly classify responders and non-responders with more than 85% probability [29]. The correlation coefficients between analytes at baseline in responders and non-responders in the current study suggest that weaker correlations between the studied proteins in lithium nonresponders at baseline may be used as a biomarker for lithium nonresponse. In contrast, stronger correlations between the analytes at baseline may be used as a biomarker for lithium response. However, a panel of proteins with defined minimal and/or maximal correlation coefficients must be determined with large sample studies in order for correlation coefficients to be used as a biomarker(s) for lithium responsiveness.

The higher levels of multi-intracellular proteins and weaker correlations between the studied proteins in lithium nonresponders at baseline suggest that the studied proteins in CD4^+^ lymphocytes and monocytes of bipolar lithium nonresponders were not functionally associated well with each other compared to those of lithium responders. Since correlation/association is not causation, how the weaker correlation in nonresponders at baseline [Figure 1] affects the higher expression levels of analytes at baseline in non-responders [16] remained unclear. The lack of association between the studied proteins might be the reason for higher levels of individual proteins in lithium non-responders. The higher levels of those proteins in nonresponders at baseline might be a sign of “compensation” or “overreaction” to some key pathological proteins/pathways. If the high levels of baseline analytes were a compensatory effect to low-level pathological proteins/pathways, the weaker correlations between the proteins suggested that the studied proteins in the network were not strongly associated with each other for this effort. Similarly, if the high levels of baseline analytes were an overreactive effort to pathological proteins/pathways, the weaker correlations between the proteins suggested that the studied proteins were not strongly associated with each other for this effort either. The decrease in almost all studied proteins after lithium in lithium nonresponders in our previous analysis [16] supports the idea that lithium is able to downregulate multiple proteins in lithium nonresponders. The downregulation may be involved in changes in associations between the studied proteins. However, we only had four patients with pre- and post-lithium blood samples in lithium nonresponders, and we were unable to see the changes in correlation coefficients in nonresponders after lithium. Correlation data between proteins after lithium in nonresponders may shed light on whether the higher levels of baseline proteins were a compensatory or an overactive effort due to a lack of “cohesive or intact” PPI networks/subnetworks. Future large sample studies with PPI network and pathway analyses may identify key pathological proteins/pathways associated with lithium nonresponse. Through manipulating the expression of some key proteins in vivo and in vitro, the mechanism of lithium nonresponse may be discovered.

In contrast to lithium nonresponders, the lower levels of most baseline analytes and stronger correlations between analytes may be used as a biomarker for lithium response. The correlation coefficients at baseline suggest that the expression levels of a majority of the analytes in responders were highly correlated (Figure 2) and functionally “connected” (Figure 3 and Figure 4). However, similar to non-responders, how the higher correlations at baseline [Figure 1] affect the low expression levels of analytes at baseline in responders [16] also remained unclear. The large number of decreased correlations between analytes (Figure 2) and increased levels of almost all analytes after lithium treatment [16] suggest that lithium may be able to “fine-tune” different pathways in lithium responders towards an optimal level. The increased levels of proteins [16] and weaker correlations between proteins after lithium treatment in responders [Figure 2] appeared contradictory. However, stronger correlations between proteins at baseline might be an indication of a “collective” effort to compensate or overly react to the abnormal levels of key pathological proteins/pathways. In contrast, the weaker correlations after lithium might be an indication of key proteins/pathways becoming “fully functioning” without the need for “non-essential” proteins/pathways. The high correlations at baseline in responders suggest that a “not fully functional but necessary” network existed for lithium response. However, it is still unclear which protein(s)/pathway(s) triggers the high functional connectivity and correlations between proteins in studied cells. Since the studied proteins were in a network of more than 130 proteins (Figure 3), it is not surprising to see a change in correlations between proteins after lithium treatment.

Previously, we found that the magnitude difference in the levels of those 28 analysis between responders and nonresponse varied widely [16]. Likely, using animal models to manipulate “key” suspected proteins and study the correlations between proteins after manipulation may help us understand a trigger(s) that affects protein functional connections. PPI network/subnetwork analyses may facilitate the finding of “key” proteins for responders (Figure 3 and Figure 4) in future studies.

The decrease in correlation coefficients between most analytes in responders after lithium is indicative of disruption to coregulatory mechanisms and could involve “fine-tuning” of intracellular protein expressions of studied blood cells. A similar process of “fine-tuning” of intracellular protein expression in neurons of the brain might also occur. However, the differences in changes of correlation coefficients of the same analytes before and after lithium in different cell types (Figure 2) suggest that even if the “fine-tuning” effect of lithium treatment in neurons of the brain occurred, the changes in the measured proteins and their correlations could be quite different from peripheral blood cells and among neurons in the different areas of the brain. The correlations between PDEB4 and other proteins (phospho-Fyn/phospho-Yes, TNFAIP3, and phospho-GSK3α/β) became stronger in CD4^+^ lymphocytes after lithium (see supplemental text and Figure S1A), but not in monocytes, support this speculation. The immune system and mitochondrial abnormality are believed to be involved in systematic toxicity in BD [7,28,40,41,42,43,44,45]. Likely, the eventual outcome of “fine-tuning” with lithium is to normalize some, if not all, abnormalities of the immune system, mitochondrial oxidative process, and other pathophysiological processes in peripheral blood cells and neurons in the central nervous system [7,29,45].

Since all patients in the current study were symptomatic, the results from this study suggest that “molecular subtypes” of bipolar disorder to lithium response may exist. In the human induced pluripotent stem cell (iPSC) study, it was found that cells from lithium responders responded to lithium, and cells from lithium nonresponders did not respond to lithium but responded to lamotrigine [8]. A recent study using a network-based multi-omics analysis found that lithium response was associated with the molecules related to focal adhesion on axon guidance and neuronal circuits [13]. Future studies using correlation coefficients between proteins, levels of proteins, PPI network/subnetwork, and pathway analyses may find the key proteins/pathways to determine lithium treatment responsiveness.

Although the correlation data suggest that an inherent regulatory difference between lithium responders and non-responders exists, and lithium seems to regulate a coregulated network of proteins in responders, as indicated by the decrease in the number and strength of correlation after treatment for this group, it should be cautious about interpreting these findings. It should be kept in mind that this study was not a hypothesis-testing study. Therefore, it should not draw any inferences from the study. The small sample size and lack of adjustment for multiple comparisons could further confound the findings. The results related to significant differences between the analytes with multiple comparisons might be incorrect or unreliable. Nevertheless, using correlation coefficients, PPI network/subnetwork, and pathway analyses to study the possible mechanism of lithium treatment responsiveness appears to be a workable concept. However, all assumptions and speculations in the current study need to be tested with large studies after the utility of this workable concept is validated.

## 5. Limitations

In addition to the small sample size, we only had four lithium non-responders with pre- and post-lithium blood samples available. We were unable to provide correlation coefficients between studied proteins in the nonresponders after lithium treatment, which limited our interpretation of the effect of lithium on the correlation between analytes in the nonresponders. Without the information on the difference in changes in correlation coefficients between analytes before and after lithium in nonresponders, we were unable to determine if the downregulation of studied proteins in nonresponders [16] was associated with an increase in correlation coefficients between the studied proteins.

The low blood collection rate at the end of the study In non-responders might be due to the lack of next treatment for patients who failed lithium treatment in the protocol, although we did offer routine clinical care for 3 months at no cost. The 16-week study duration for the current study might be too long for those who did not have much benefit from lithium monotherapy. A shorter study duration with a contingency treatment plan for those who do not have much benefit from lithium monotherapy in future studies may increase the rate of blood collection from lithium non-responders at the end of the study.

Although the comparability of blood and brain have been investigated at different levels with an overall impression that DNA methylation level, gene expression patterns, and some protein changes are similar between brain and blood cells [46,47,48,49], differential correlations in CD4^+^ lymphocytes and monocytes suggest that the different neurons in the brain can be affected by lithium in different ways. As mentioned earlier, using the changes in blood cells is unlikely to reflect the same changes in the brain.

## 6. Conclusions

The results from this post-hoc analysis suggest that using correlation coefficients, PPI network and subnetwork, and pathway analysis with multiple intracellular proteins in the CD4^+^ lymphocytes and monocytes of bipolar patients may help us study the mechanism of lithium treatment responsiveness. However, large sample size studies are necessary to determine the utility of this approach before it can be considered for future hypothesis testing studies.

## Figures and Tables

**Table 1:** Results of Pathway Enrichment Analysis and Genes Involved in Pathways Based on the Protein-Protein Networks of the 23 Proteins.

Pathway	Genes	*p*-Value
Prolactin Signaling Pathway	***FYN*; *GSK3β*; *MTOR*; *NFKB*; *IRS2*;** *ITGB1*; *IRS1*; *SHC1*; *STAT3*; *IRS2*; *PTPN11*; *PIK3R1*; *YWHAZ*; *VAV1*; *ERBB2*; *GRB2*; *PTPN6*; *RAF1*	1 × 10^26^
Leptin signaling pathway	***FYN*; *GSK3β*; *NFKB*; *MTOR*; *IRS1*;** *SHC1*; *STAT3*; *PTPN11*; *PIK3R1*; *ESR1*; *SP1*; *ERBB2*; *GRB2*; *RAF1*	1 × 10^21^
Brain-Derived Neurotrophic Factor (BDNF) signaling pathway	***GSK3β*; *IRS2*; *MTOR*; *NFKB*; *FYN*; *BDNF*;** *NTRK2*; *NCF1*; *IRS1*; *SHC1*; *PRKCD*; *STAT3*; *PTPN11*; *PIK3R1*; *SYN1*; *CTNNB1*; *GRB2*; *MAPT*; *RAF1*	1 × 10^25^
Neurotrophin signaling pathway	***CALM1*; *IRS2*; *BCL2*; *BDNF*; *GSK3β*; *NFKB*;** *YWHAE*; *NTRK2*; *IRS1*; *SHC1*; *PRKCD*; *PTPN11*; *PIK3R1*; *YWHAZ*; *YWHAQ*; *ABL1*; *GRB2*; *RAF1*; *TP53*; *YWHAH*	1 × 10^28^

Note: The bold genes are implicated in the current study. Abbreviations: BCL2: B-cell lymphoma 2; BDNF: Brain-derived neurotrophic factor. CALM1: calcium-modulated protein. FYN: a tyrosine kinase belongs to the Src family of tyrosine kinases, including src, fyn, and yes; GSK3β: Glycogen synthase kinase 3 beta: IRS2: insulin receptor substrate; MTOR: Mammalian target of rapamycin; NFKB: nuclear factor NF-kappa-B p65 subunit.

## Data Availability

Data are available upon request.

## Supplementary Materials

The following supporting information can be downloaded at: https://www.mdpi.com/article/10.3390/jcm13051491/s1, Figure S1. Correlation coefficient R squared between PDEB4 and other analytes before and after lithium in CD4^+^ lymphocytes (A) and monocytes (B) in lithium responders. Figure S2. Correlation coefficient R squared between NLRP3 and other analytes before and after lithium in CD4^+^ lymphocytes (A) and monocytes (B) in lithium responders. Figure S3. A protein-protein interaction network of 23 studied proteins related to the 28 analytes in monocytes that are mapped to the BioGRID database.
