# Supplementary material for: Possible Role of Correlation Coefficients and Network Analysis of Multiple Intracellular Proteins in Blood Cells of Patients with Bipolar Disorder in Studying the Mechanism of Lithium Responsiveness: A Proof-Concept Study
Source: J Clin Med. 2024 Mar 5;13(5):1491. doi: 10.3390/jcm13051491 (PMC10935410; doi:10.3390/jcm13051491)
Supplement: Supplementary file 1 [file jcm-13-01491-s001.zip › jcm-2825433-supplementary.pdf]

Supplemental materials

Correlation between PDEB4 and analytes before and after lithium treatment in responders

Since PDEB4 was significantly increased with lithium treatment in both lymphocytes and monocytes [1], the correlation of its expression levels with other analytes before and after lithium was estimated with correlation coefficient. In lymphocytes (supplemental Figure S1A), before lithium (blue bar), the correlation coefficients between PDEB4 and other analytes varied widely The levels of the majority of analytes had significant correlations with PDEB4 with 19 analytes having a p values of ≤ 0.05 and 10 analytes (GSK3β, NLRP3, phospho-CREB, PPAG-γ, TNFAIP3, PKC-θ, PKA-C-α, BDNF, Fyn, PGM1) having a p value of ≤0.01. After lithium (brown bar), the correlation coefficients between PDEB4 and other analytes still varied widely (Figure S1A), and the levels of 12 analytes were still significantly correlated to the level of PDEB4 with a p value of ≤ 0.05 and 5 analytes (BAK, phospho-Fyn/yes, phospho-CREB, TNFAIP3, TPH1) were still significant with a p value of ≤0.01.

In monocytes (supplemental Figure S1B), before lithium (blue bar), the correlation coefficients between PDEB4 and other analytes varied widely The levels of 10 analytes had significant correlations with PDEB4 with a p values of ≤ 0.05 and 8 analytes (GSK3β, NLRP3, phospho-CREB, PPAG-γ, PKA-C-α, BCL-2A1, MARCKS, Fyn) having a p value of ≤0.01. After lithium (brown bar), the correlation coefficients between PDEB4 and other analytes still varied widely, and the levels of 4 analytes (BCL-2A1, phospho-Fyn/yes, phospho-GSK3αβ, PGM1) were still significantly correlated to the level of PDEB4 with a p value of ≤ 0.05. However, none was still significant with a p value of ≤0.01.

Correlation coefficients between NLRP3 and other analytes in responders before and after lithium treatment

NLRP3 did not increase significantly after lithium treatment. The correlation of NLRP3 expression level with other analyte levels in completed responders before and after lithium was estimated with correlation coefficients as GSK3β with other analytes. In lymphocytes supplemental Figure S2A), before lithium (blue bar), the correlation coefficients between NLRP3 and other analytes varied widely. The levels of the majority of analytes had significant correlations with NLRP3 with 15 analytes having a p values of ≤ 0.05 and 9 analytes (PDEB4, phospho-CREB, TNFAIP3, PKC-θ, PKA-C-α, BCL2-A1, BDNF, Fyn, timeless) having a p value of ≤0.01. After lithium (brown bar), the correlation coefficients between NLRP3 and other analytes still varied widely, and the levels of 2 analytes were still significantly correlated to the level of NLRP3 with a p value of ≤ 0.05 (TNFAIP3, BDNF).

In monocytes (supplemental Figure S2B), before lithium (blue bar), the correlation coefficients between NLRP3 and other analytes varied widely. The levels of 7 analytes had significant correlations with PDEB4 with a p values of ≤ 0.05 (PDEB4, phospho-CREB, PPAR-γ, PKA-C-α, BCL2-A1, MARCKS) and 1 analytes (PDEB4) having a p value of ≤0.01. After lithium (brown bar), the correlation coefficients between PDEB4 and other analytes still varied widely, and the levels of 2 analytes with a p values of ≤ 0.05 (phospho- GSK3β and phospho-GSK3α). However, none was still significant with a p value of ≤0.01.

Protein-Protein Interaction Network Analysis

Of the 28 analytes, there were 23 proteins. The PPI network of these 23 proteins in monocytes was generated by using the same methodology as previously described [2]. These 23

proteins are in a network of more than 130 proteins in CD4$^+$ lymphocytes (see Figure S3) and monocytes (see supplemental Figure S3). All studied proteins in the network had a lower level of expression in lithium responders than in nonresponders.

References:

1. Gao, K.; Kaye, N.M.; Ayati, M.; Koyuturk, M.; Calabrese, J.R.; Christian, E.; Lazarus, H.M.; Kaplan, D. Divergent Directionality of Immune Cell-Specific Protein Expression between Bipolar Lithium Responders and Non-Responders Revealed by Enhanced Flow Cytometry. *Medicina* 2023, *59*, 120.

2. Gao, K.; Ayati, M.; Koyuturk, M.; Calabrese, J.R.; Ganocy, S.J.; Kaye, N.M.; Lazarus, H.M.; Christian, E.; Kaplan, D. Protein Biomarkers in Monocytes and CD4$^+$ Lymphocytes for Predicting Lithium Treatment Response of Bipolar Disorder: A Feasibility Study with Tyramine-Based Signal-Amplified Flow Cytometry. *Psychopharmacol. Bull.* 2022, *52*, 8–35.

Supplemental Figure S1. Correlation coefficient R squared between PDEB4 and other analytes before and after lithium in CD4+ lymphocytes (A) and monocytes (B) in lithium responders

A.

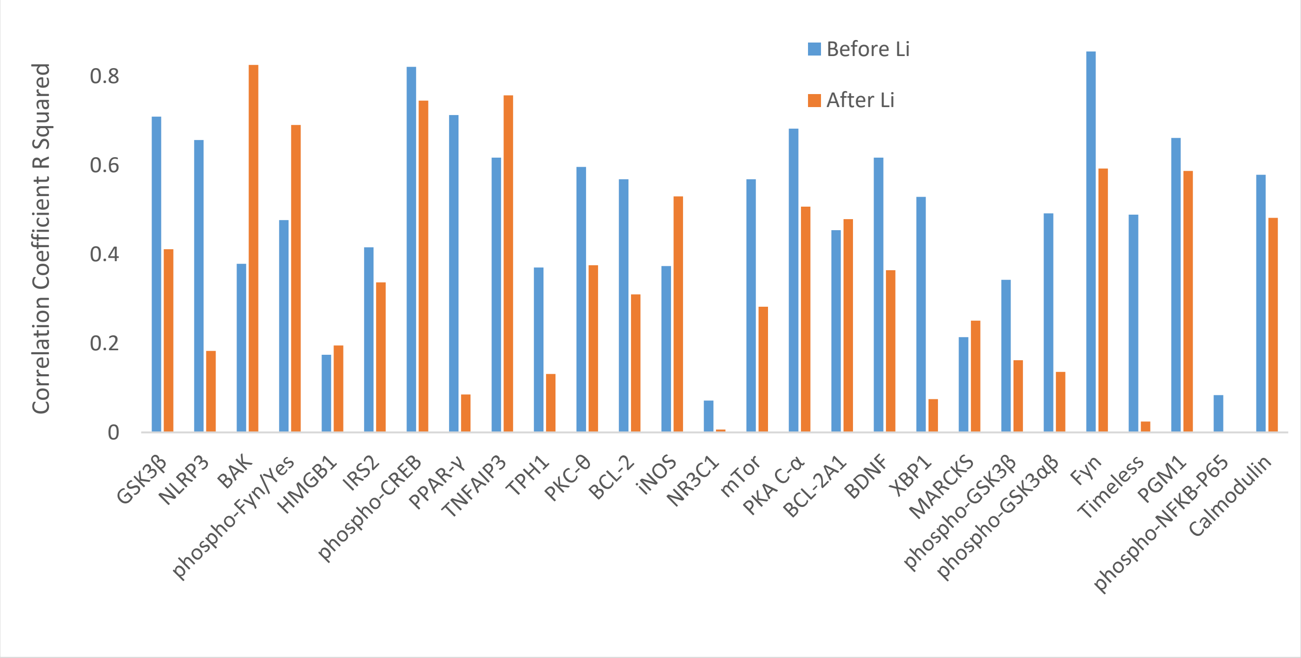

B.

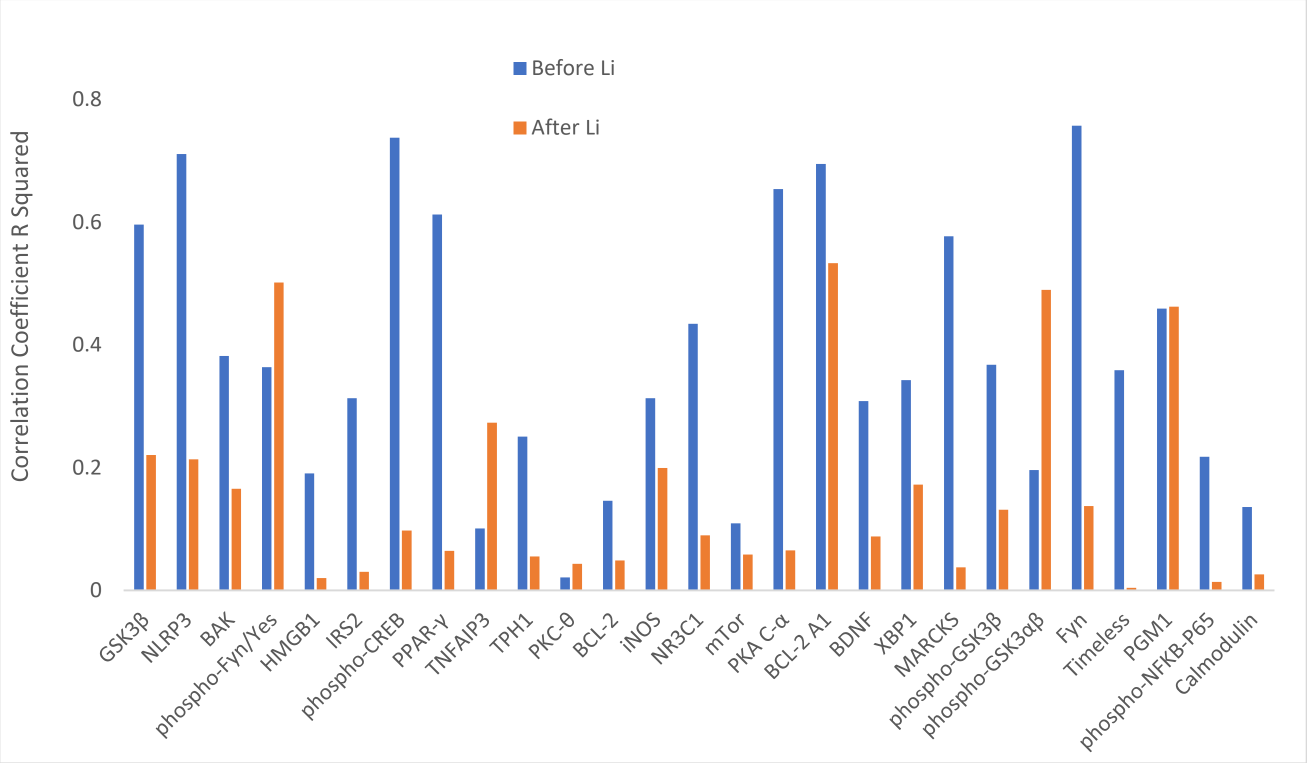

Abbreviations: BAK: BAX, BCL2-Associated X Protein; BCL-2: B-cell lymphoma 2; BCL-2 A1: Bcl-2-related protein A1; BDNF: brain-derived neurotrophic factor; Calmodulin: calcium-

modulated protein; Fyn: a tyrosine kinase belongs to the Src family of tyrosine kinases including src, fyn, and yes; GSK-3β: glycogen synthase kinase 3 beta; HMGB1: High mobility group box 1 protein; iNOS: inducible isoform nitric oxide synthases; IRS2: Insulin receptor substrate 2; MARCKS: myristoylated alanine-rich C-kinase substrate; mTor: mammalian target of rapamycin; NLRP3: NACHT, LRR and PYD domains-containing protein 3; NR3C1: nuclear receptor subfamily 3, group C, member 1; phospho-CREB: phosphorylated cAMP response element-binding protein (Ser133); phospho-Fyn /Yes: phosphorylated Fyn(Y530)/Yes(Y537); phospho-GSK 3 α/β: phosphorylated glycogen synthase kinase 3 alpha(Tyr279) beta(Tyr216); phospho- GSK 3β: phospho-glycogen synthase kinase 3 beta(Tyr216); phospho-NFKB-P65: phosphorylated nuclear factor NF-kappa-B p65(Ser536) subunit; PDEB4: cAMP-specific 3',5'-cyclic phosphodiesterase 4B; PGM1: phosphoglucomutase 1; PKA C-α: protein kinase A catalytic subunit; PKC-θ: protein kinase C theta; PPAR-γ: peroxisome proliferator-activated receptor gamma; Timeless: a protein is necessary of proper functioning of circadian rhythm; TNFAIP3: tumor necrosis factor, alpha-induced protein 3; TPH1: tryptophan hydroxylase 1; XBP1: X-box binding protein 1.

Supplemental Figure S2. Correlation coefficient R squared between NLRP3 and other analytes before and after lithium in $CD4^+$ lymphocytes (A) and monocytes (B) in lithium responders

A.

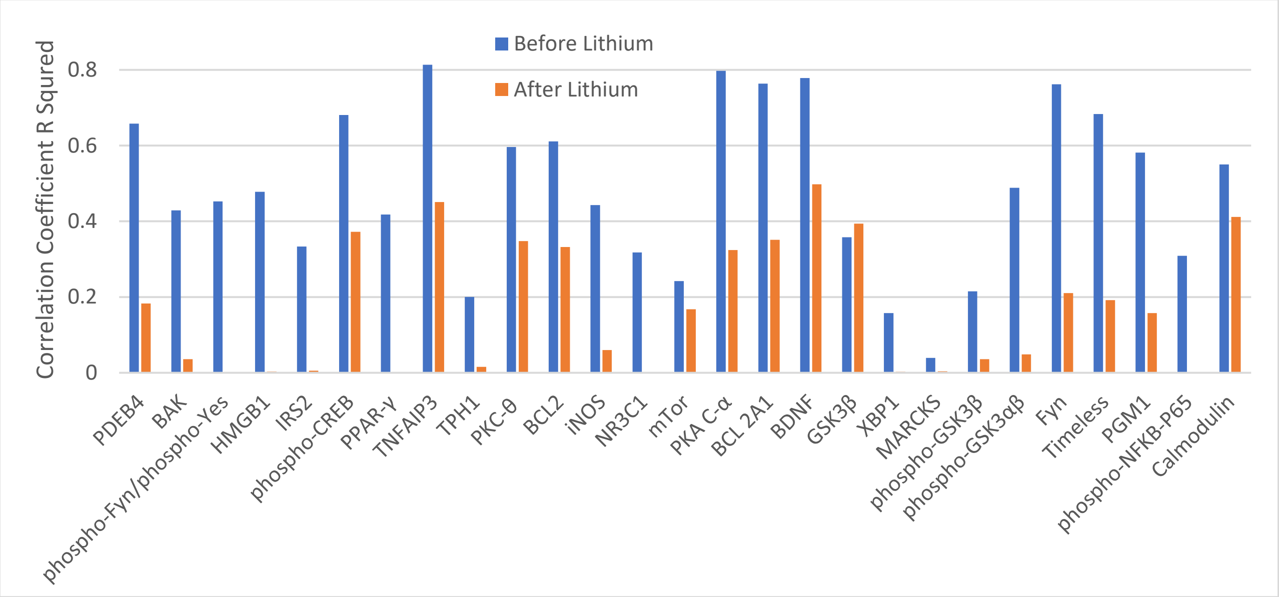

B.

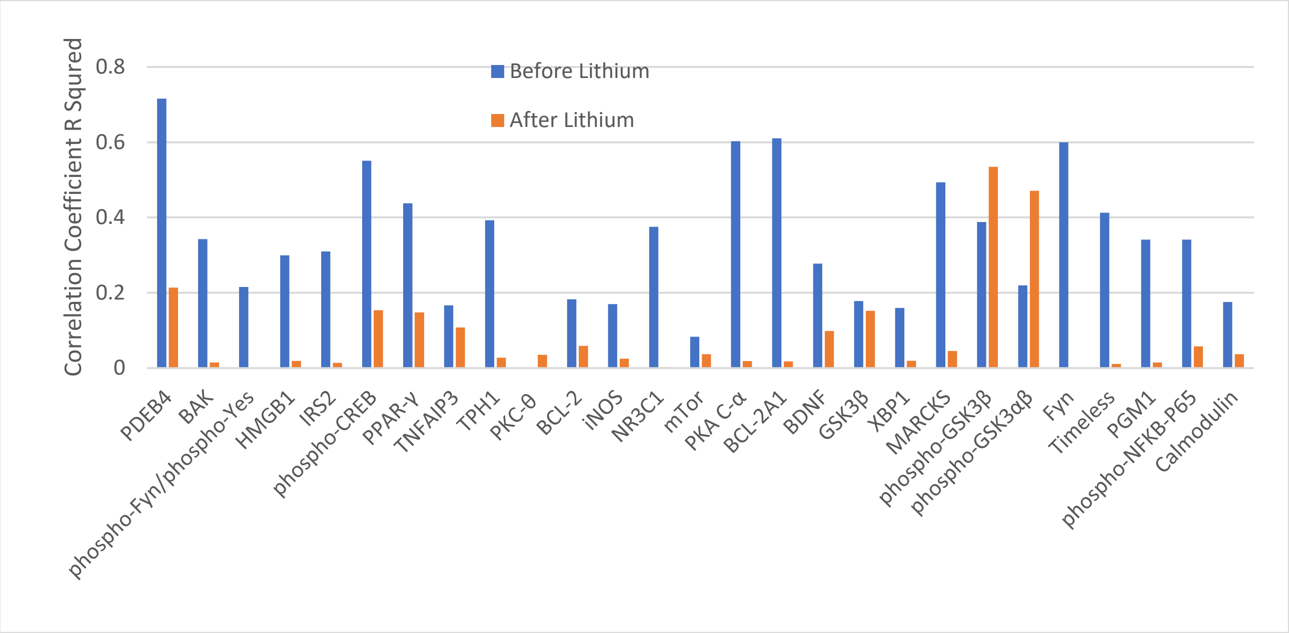

Abbreviations: BAK: BAX, BCL2-Associated X Protein; BCL-2: B-cell lymphoma 2; BCL-2 A1: Bcl-2-related protein A1; BDNF: brain-derived neurotrophic factor; Calmodulin: calcium-modulated protein; Fyn: a tyrosine kinase belongs to the Src family of tyrosine kinases including src, fyn, and yes; GSK-3β: glycogen synthase kinase 3 beta; HMGB1: High mobility group box 1 protein; iNOS: inducible isoform nitric oxide synthases; IRS2: Insulin receptor substrate 2;

MARCKS: myristoylated alanine-rich C-kinase substrate; mTor: mammalian target of rapamycin; NLRP3: NACHT, LRR and PYD domains-containing protein 3; NR3C1: nuclear receptor subfamily 3, group C, member 1; phospho-CREB: phosphorylated cAMP response element-binding protein (Ser133); phospho-Fyn /Yes: phosphorylated Fyn(Y530)/Yes(Y537); phospho-GSK 3 α/β: phosphorylated glycogen synthase kinase 3 alpha(Tyr279) beta(Tyr216); phospho- GSK 3β: phospho-glycogen synthase kinase 3 beta(Tyr216); phospho-NFKB-P65: phosphorylated nuclear factor NF-kappa-B p65(Ser536) subunit; PDEB4: cAMP-specific 3',5'-cyclic phosphodiesterase 4B; PGM1: phosphoglucomutase 1; PKA C-α: protein kinase A catalytic subunit; PKC-θ: protein kinase C theta; PPAR-γ: peroxisome proliferator-activated receptor gamma; Timeless: a protein is necessary of proper functioning of circadian rhythm; TNFAIP3: tumor necrosis factor, alpha-induced protein 3; TPH1: tryptophan hydroxylase 1; XBP1: X-box binding protein 1.

Supplemental Figure S3. A protein-protein interaction network of 23 studied proteins related to the 28 analytes in monocytes that are mapped to the BioGRID database.

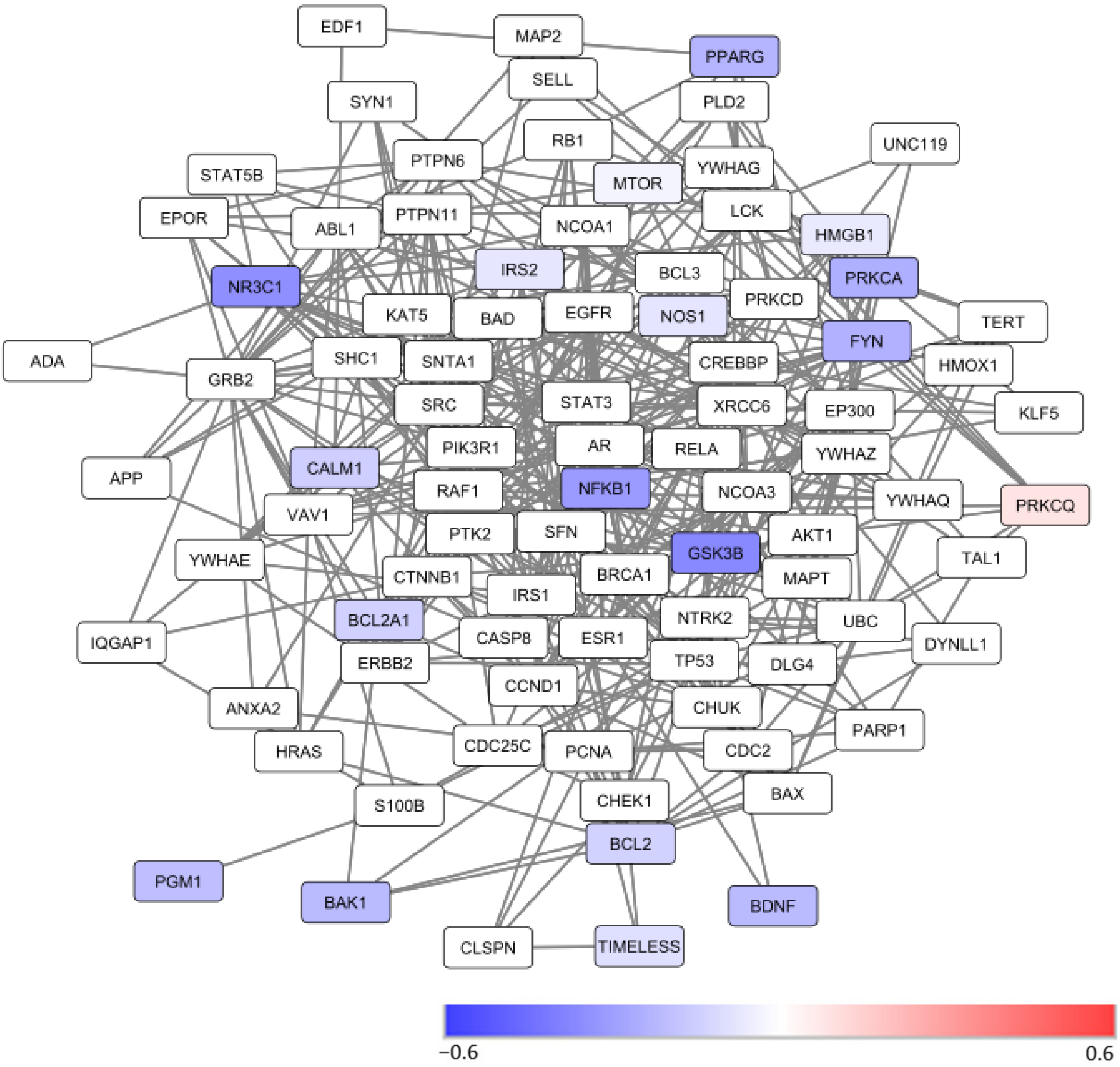

Note: The color of the nodes represents the magnitude of differences in protein expression levels between lithium responders and non-responders at the baseline as measured with the $\log_2(\frac{the\ average\ of\ MFR\ of\ Responders}{the\ average\ of\ MFR\ of\ Non\text{−}Responders})$. A positive value is indicative of a higher level of protein expression in lithium responders relative to non-responders. A negative value is indicative of a lower level of protein expression in lithium responders relative to non-responders. The white nodes are proteins that were not measured in the current study, but they are on the shortest paths between pairs of proteins that are included in the study. PDEB4 was not in the network; phospho-GSK3β, GSK3β, and phospho-GSK3αβ = GSK; fyn and phospho-fyn/yes = FYN. Abbreviations: BAX1: BAX, BCL2-Associated X Protein; BCL2: B-cell lymphoma 2; BCL2A1: Bcl-2-related protein A1; BDNF: Brain-derived neurotrophic factor; CALM1:

calcium-modulated protein; CREB1: phosphorylated cAMP response element-binding protein; FYN: a tyrosine kinase belongs to the Src family of tyrosine kinases including src, fyn, and yes; GSK3B: Glycogen synthase kinase 3 beta; HMGB1: High mobility group box 1 protein; IRS2: insulin receptor substrate; MARCKS: myristoylated alanine-rich C-kinase substrate; MTOR: Mammalian target of rapamycin; NLPR3: NACHT, LRR and PYD domains-containing protein 3; NOS1: Inducible isoform nitric oxide synthase; NR3C1: nuclear receptor subfamily 3, group C, member 1; PGM1: Phosphoglucomutase 1; PPARG: Peroxisome proliferator-activated receptor gamma; PRKCA: Protein kinase A catalytic subunit alpha; PRKCQ: protein kinase C theta; NFKB1: nuclear factor NFkB p65 subunit; TIMELESS: a protein is necessary of proper functioning of circadian rhythm; TNFAIP3: tumor necrosis factor, alpha-induced protein 3; TPH1: Tryptophan hydroxylase 1.
